# Minimally Invasive Far Lateral Lumbar Discectomy With Modified Technique: Symptomatic Relief and Intersegmental Stability Study

**DOI:** 10.7759/cureus.53415

**Published:** 2024-02-01

**Authors:** Nicholas M Laskay, Matthew T Jarrell, Arsalaan Salehani, Travis Atchley, Matthew S Parr, James Mooney, Nicholas J Erickson, Sasha Howell, Mamerhi Okor, Daniel Harmon

**Affiliations:** 1 Neurological Surgery, University of Alabama at Birmingham School of Medicine, Birmingham, USA; 2 Medicine, University of Alabama at Birmingham School of Medicine, Birmingham, USA

**Keywords:** surgery spine, minimally invasive spine surgery, lumbar discectomy, far lateral disc herniation, extra-foraminal disc herniation

## Abstract

Objective: To evaluate the use of a modified minimally invasive surgery (MIS) technique for far lateral lumbar discectomy (FLDH) that minimizes the degree of bony drilling required for nerve root decompression, increasing postoperative pain reduction rate with reduced risk of iatrogenic spinal instability.

Summary of background data: FLDH accounts for approximately 10% of all lumbar disc herniations and is increasingly recognized in the era of advanced imaging techniques. These disc herniations typically result in extra-foraminal nerve root compression. Minimally invasive spine techniques are increasingly performed with various degrees of foraminal and facet removal to decompress the affected nerve root.

Methods: The study design involves a single institutional, retrospective cohort technical review. The review was completed of all patients undergoing MIS far lateral lumbar discectomy between 2010 and 2020. Cross-sectional, summary statistics were calculated for all variables. Counts and percentages were recorded for categorical variables and mean and standard deviations were calculated for continuous variables.

Results: A total of 48 patients underwent MIS far lateral lumbar discectomies (FLLD) from 2010 to 2020. The mean age was 63 ± 11.5 years (60.4% males), the mean BMI was 28.5 ± 5.5, and 20.8% smokers. The most common presenting complaint was both low back and radicular pain (79.2%) with 8.3% of patients suffering from motor weakness preoperatively. The mean follow-up time was 4.3 ± 2.7. The mean length of stay was 1.3 ± 1.4 days with 77.1% of patients discharged postoperative day one. Forty-three patients (93.5%) had improvement in their symptoms. Twenty-seven (58.7%) had complete resolution in 2.6 months on average. Six patients (13%) had immediate symptom resolution postoperatively.

Conclusions: Our modified technique for FLLD allows MIS access to the extra-foraminal site of nerve root compression without the need for bony drilling. This minimizes postoperative pain and reduces the risk of iatrogenic spinal instability without sacrificing symptom resolution.

## Introduction

Far lateral lumbar disc herniations (FLDH) account for approximately 7-12% of all lumbar disc herniations [1-3]. These disc herniations result in extra-foraminal nerve root compression or less frequently foraminal compression. Unlike medial disc herniations, lateral discs tend to compress the nerve exiting at the same interspace rather than the traversing nerve root [4-7]. The lumbar 4-lumbar 5 (L4-5) level is the most commonly affected and patients often present with radicular pain in the hip and leg in addition to quadriceps weakness, altered thigh/leg sensation, and/or decreased patellar reflexes [8].

Various techniques for far lateral discectomy have previously been described, and minimally invasive surgery (MIS) techniques are increasingly being performed [2,6,9,10]. MIS techniques are evolving and increasingly utilized as they are less destabilizing to the spinal column and often provide more direct access to surgical pathology [2,4,9,11].

The traditionally described MIS technique usually involves a paramedian incision with the insertion of dilators and tubular retractors with an ideal working view of the inferior aspect of the superior level transverse process (TP), lateral aspect of the superior level pars, the facet joint, and the intertransverse space. Soft tissue and bony decompression then follow as necessary for nerve root decompression. Authors report various degrees of foraminal and facet drilling to expose and decompress the affected nerve root [6,12,13]. At our institution, we attempt to minimize the degree of bony decompression and prefer non-drill techniques to reduce potential risks to the soft tissue and neural elements. We hypothesize this may also result in less postoperative pain and reduced risk of iatrogenic spinal instability. Here, we describe a modified technique for MIS tubular-assisted discectomy for nerve root decompression. We also report our single-institution experience of patients with FLDH undergoing this modified technique. Portions of this study were orally presented at the Spine Summit in San Diego, United States, July 28-31, 2021.

## Materials and methods

A retrospective cohort study was conducted through a chart review of all consecutive patients undergoing MIS lumbar far lateral discectomy by a single surgeon between January 1, 2010, and December 31, 2020. Patients were identified using administrative billing data. University of Alabama at Birmingham Hospital Institutional Review Board approval (300009309) was obtained prior to the study. Demographic, preoperative, and postoperative data for each patient were collected via review of the electronic medical record (EMR). All patients 18 years of age or older undergoing elective single-level or multi-level MIS far lateral lumbar discectomy for radiculopathy with or without back pain were included. All patients included underwent MIS discectomy using a modified version of the traditional technique. Patients requiring revision surgery as the index procedure were excluded. This manuscript complies with the Strengthening the Reporting of Observational Studies in Epidemiology (STROBE) checklist for cohort studies [14].

Perioperative outcomes

**Figure 1:**
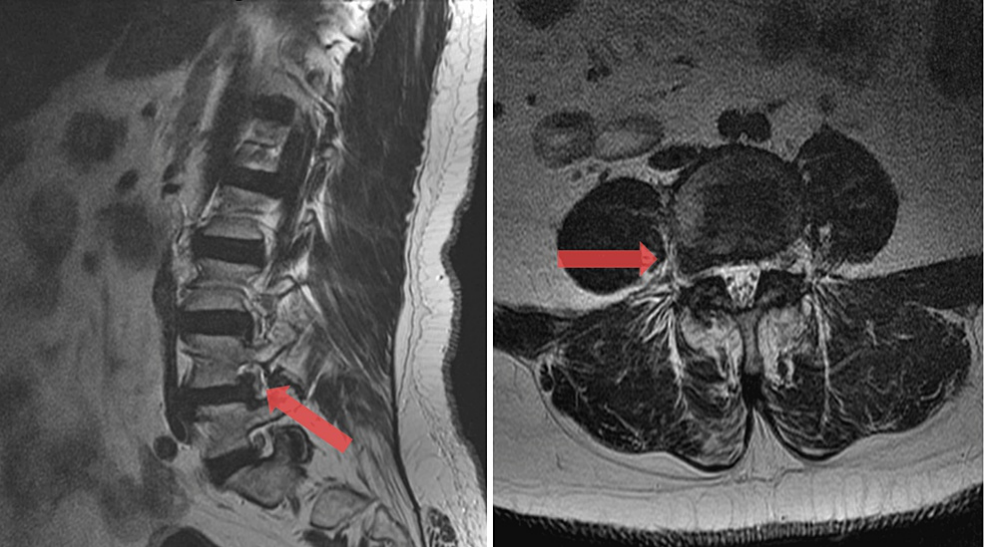
Far lateral disc herniation T2 weighted magnetic resonance imaging, sagittal (left) and axial (right). Red arrow depicting right-sided extra-foraminal L4 nerve root impingement from far lateral disc herniation.

Presenting symptoms were defined as isolated low back pain (LBP), LBP with radicular pain, isolated radicular pain or radicular pain with weakness, or a combination of LBP with radiculopathy based on clinical exam at the time of presentation. The level of disc herniation was defined on preoperative magnetic resonance imaging (MRI) as a single intervertebral disc and its cranial and caudal vertebral body (Figure 1). All patients had previously failed aggressive medical management including non-steroidal anti-inflammatory drugs, physical therapy, and epidural steroid injection (ESI). Patients with lingering or return symptoms received a postoperative MRI for assessment of nerve root decompression or return of disc herniation. In-hospital length of stay (LOS) and time course for symptom resolution were defined in days. Discharge disposition was defined as home, home with home health assistance, inpatient rehab, or skilled nursing facility. The need for postoperative ESI and/or revision surgery was made at the discretion of the neurosurgeon at follow-up.

Statistical analysis

Counts and percentages were recorded for categorical variables (race, sex, presenting symptoms, degree of symptom improvement postoperatively, discharge disposition, and need for additional intervention), and means and standard deviations were calculated for continuous variables (age, length of surgery, hospital LOS). All continuous variables were normally distributed. Statistical comparison to any historical or contemporaneous cohort of traditional far-lateral discectomy or natural history was outside of the scope of the current investigation and therefore was not conducted. Stata MP 14.0 (StataCorp, College Station, United States) was used for summary statistics.

Operative technique

**Figure 2:**
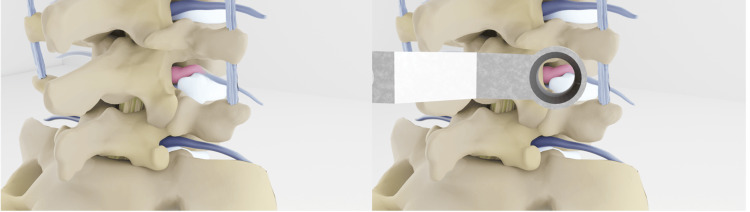
Right L4-5 far lateral disc herniation A) Right L4-5 far lateral disc herniation with compression and displacement of the L4 nerve root cranially. B) Access of the extraforaminal disc herniation by identification of the inferior right L4 transverse process, pars interarticularis, L4-5 facet joint, and L4-5 interspace. Credits: Michael Cloyde

Upon arrival to the operating room, the patient is placed prone on a Wilson frame on top of a flat Jackson table. Under fluoroscopic guidance, a spinal needle is used to localize the lumbar level of interest. A 2cm incision is made 1.5cm off midline overlying the interspace on the side of the far lateral disc herniation. A guidewire, sequential dilators, and ultimately a tubular retractor are used to gain access to the requisite anatomy. For example, in a right L4-5 far lateral disc herniation, the inferior right L4 TP, lateral right L4 pars, right L4-5 facet joint, and right L4-5 intertransverse space are accessed (Figure 2).

**Figure 3:**
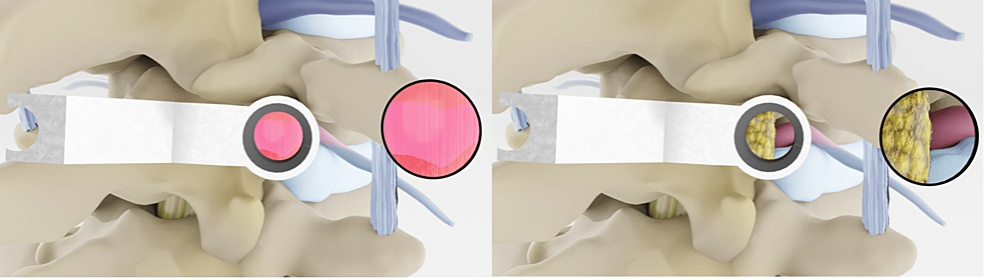
Identification of musculature and perineural fat A) Overlying and intertransverse musculature that is coagulated and resected during the minimally invasive access port approach to the disc. B) Perineural fat is identified and carefully evacuated allowing extraforaminal vasculature to be identified, coagulated, and ligated. Credits: Michael Cloyde

**Figure 4:**
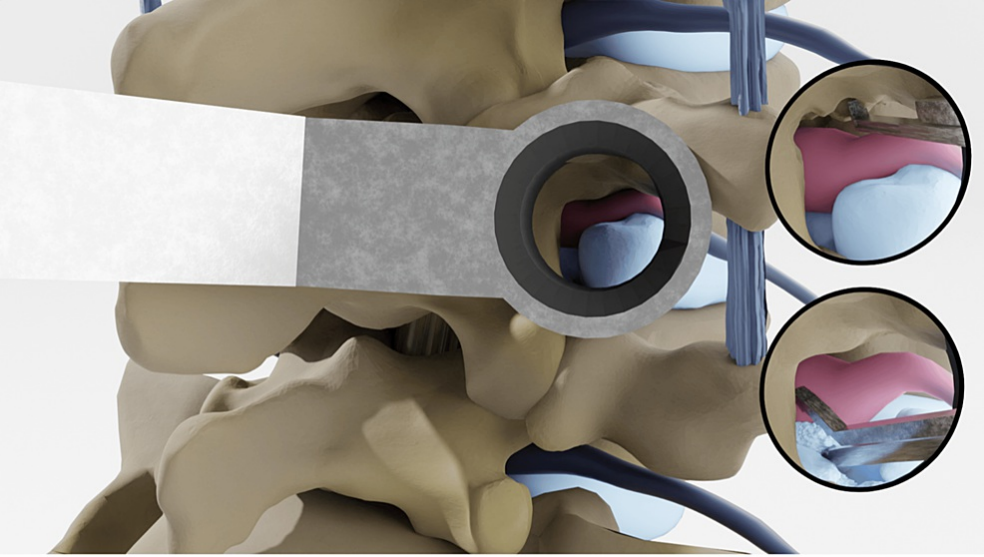
Foraminal decompression The exiting nerve root is then tracked back to the foramen. Portions of the foramen are resected with a Kerrison punch (A) and the disc is removed carefully (B). Credits: Michael Cloyde

Unipolar electrocautery is used for soft tissue dissection exposing the landmarks above. The overlying and intertransverse musculature is coagulated and resected exposing the intertransverse fascia. Resection of this fascia reveals the perineural fat, which is carefully evacuated with suction allowing extraforaminal vasculature to be identified, coagulated, and ligated (Figure 3). The target nerve root is then identified within the foraminal compartment and mobilized to access the offending disc herniation. The disc protrusion is carefully dissected off the compressed nerve root prior to incising the disc annulus and resecting disc fragments piecemeal (Figure 4). This is continued until the ventral surface of the compressed nerve root is adequately decompressed within the subarticular compartment. The nerve root is then tracked back towards its foramen to ensure adequate decompression. If necessary, portions of the lateral aspect of the foramen are removed to achieve adequate foraminal decompression (Figure 3). Depo-medrol is placed into the extra-foraminal compartment bathing the previously compressed nerve root. Hemostasis is then achieved followed by the removal of the tubular retractor and closure of the skin incision.

## Results

A total of 48 consecutive patients underwent MIS lumbar far lateral discectomies from 2010 to 2020. The mean age was 63 ± 11.5 years old with 60.4% males, a mean BMI of 28.5 ± 5.6, and 20.8% smokers. The racial breakdown was 77.8% white, 17.8% black, and 4.4% Asian (Table 1). The most common presenting complaint was both LBP and radicular pain (79.2%) followed by isolated radicular pain (18.7%), and isolated LBP (2.1%). The mean follow-up time for the entire cohort was 4.3 ± 2.7 months. Only four patients (8.3%) suffered from motor weakness preoperatively.

**Table 1:** Patient demographics *Reported as mean ± standard deviation

N=	48
Male sex	29 (60.4%)
Age^*^	63 ± 11.5
Race	
White	35 (77.8%)
Black	8 (17.8%)
Asian	2 (4.4%)
BMI^*^	28.5 ± 5.5
Current smoker	10 (20.8%)
Follow up^*^ (months)	4.3 ± 2.7

Upon MRI evaluation, 45 patients (93.8%) had foraminal or extra-foraminal disc herniations, while three (6.2%) had nerve root compression from osteophytes. The level of pathology was distributed as listed in Table 2, with the most commonly affected level being L4-5 (42%) followed by lumbar 3-4 (30%). There were two patients with herniations at two levels.

**Table 2:** Distribution of disc herniations

Level	n (%)
L1-2	1 (2%)
L2-3	4 (8%)
L3-4	15 (30%)
L4-5	21 (42%)
L5-S1	9 (18%)
*One patient had herniations at both L2-3 and L3-4, and one had herniations at L3-4 and L4-5	

No immediate postoperative complications occurred in the cohort. Mean LOS was 1.3 ± 1.4 days with 77.1% of patients discharged postoperative day one. Almost all patients (91.6%) were discharged home, while 4.2% were discharged to inpatient rehabilitation, and another 4.2% were discharged with home health physical therapy. Of the 46 patients in whom follow-up was complete, 43 patients (93.5%) had improvement in their symptoms, and 27 (58.7%) ultimately had complete resolution in 2.6 months on average (Table 3). Six patients (13%) had immediate resolution of symptoms postoperatively.

**Table 3:** Clinical outcomes at last follow-up The mean follow-up time for the entire cohort was 4.3 ± 2.7 months.

Length of stay (mean)	1.3 ± 1.4 days
Symptom improvement rate	43 (93.5%)
Complete resolution rate	27 (58.7%)
Postoperative complications rate	0 (0%)
Required additional surgical procedures	3 (6.25%)

Of the 46 patients with adequate follow-up, 37% reported residual radicular pain, 13% reported residual LBP, and 13% reported both LBP and radicular pain (Table 4). One patient (2.2%) reported numbness alone and three patients (6.5%) reported paresthesia. Two (4.3%) of the original four patients who suffered from weakness preoperatively also suffered from residual weakness postoperatively.

**Table 4:** Symptoms reported at postoperative follow-up

Symptom	n (%)
Radicular pain	17 (37.0%)
Low back pain	6 (13.0%)
Radicular + low back pain	6 (13.0%)
Numbness	1 (2.2%)
Paresthesias	3 (6.5%)
Weakness	2 (4.3%)

Fourteen patients had postoperative MRIs. Of these, only two patients had residual disc herniations identified. There was nerve root enhancement on postcontrast imaging in three of the patients with interval nerve root decompression on their imaging.

Three patients underwent additional surgical procedures after their discectomy (Table 3). One patient underwent an MIS decompression and interbody fusion for residual spondylosis and foraminal stenosis identified on postoperative MRI. Two patients underwent spinal cord stimulator placement for persistent radicular pain. Only three patients required postoperative ESI, one of whom was the previous patient who underwent subsequent redo decompression and fusion.

## Discussion

Several surgical approaches for the FLDH have been adopted and described over the last 20 years [4,10,15-17]. Conventional midline approaches often utilize a large laminotomy and partial facetectomy to expose the herniated disc for surgical removal [15]. However, this approach may lead to intersegmental destabilization of the lumbar spine and may contribute to chronic back pain postoperatively. Watkins (1953) described a paramedian approach that was later modified by Wiltse et al. (1968) that split the paraspinal musculature and preserved the facet joints to access the foraminal or extra-foraminal regions [18,19]. In the last three decades, increased awareness of motion segment instability and chronic LBP attributable to facet joint disruption has generated a growing body of literature advocating for a paraspinal approach [13,20-22]. Heterogenous methods of approaching the offending pathology while trying to preserve intersegmental stability have since been described [13,20-22].

The more recent advent of MIS surgery, which embraces the use of tubular retractors, has further enabled spine surgeons to approach the extra-foraminal region with minimal disruption of the stabilizing tissues of the lumbar spine. Several modifications of the MIS technique have since been described in the literature [4,5,9,23-27]. Epimenio et al. (2003) reported results of 46 patients with FLDHs treated with 3-4cm paramedian incision, no bony resection, and minimal muscle retraction. No complications were reported and all patients were discharged within two days without low back or radicular complaints. Eight patients had residual lower back discomfort at follow-up [12]. Quaglietta et al. (2005) described a paramedian muscle-splitting intertransverse technique on 42 patients with FLDHs. They comment on increased visualization of the exiting nerve root and herniated disc without relying on excessive bony resection. About 22 of the 25 patients who had follow-up had resolution of their radicular symptoms. 12 patients had persistent postoperative pain that improved within 15-21 days [13]. Salame and Lidar (2010) described 31 patients who underwent minimally invasive discectomies through a 15mm diameter port. About 22 out of 31 patients had resolution of their preoperative radicular pain with improvement but no resolution in five patients [26].

Contrary to conventional principles that require laminotomy and partial facetectomy, our MIS technique focuses on direct extra-foraminal nerve root decompression with little to no bony removal. Despite this, our study shows that a MIS far lateral approach with minimal bony removal is well-tolerated, safe, and can provide a high rate of symptomatic relief while preserving the posterior stabilizing elements of the lumbar spine. Our mean LOS of 1.3 ± 1.4 days is consistent with findings from other studies employing MIS techniques for far lateral disc herniation surgery, suggesting that our approach maintains the efficiency and reduced hospitalization benefits characteristic of minimally invasive spine surgeries [28,29]. In our series, almost all of the patients in the cohort had subjective improvement in their symptoms (93.5%), and more than half ultimately had complete resolution at follow-up (58.7%). However, about a third of patients (37%) still reported radicular pain in the same distribution. While this technique is successful at decompressing the offending disc at surgery, lingering radiculopathy postoperatively may underscore the difficulty in the diagnosis of FLDHs. Difficult diagnosis of FLDH even with modern imaging modalities and expert clinical examinations may delay effective treatment strategies for these patients; thus, they may present for surgical consultation later in the disease process making complete radicular resolution postoperatively a difficult task. Furthermore, two of the patients in the series (4.3%) suffered from residual distal lower extremity weakness at follow-up. It is important to note that these two patients had pre-existing weaknesses before undergoing surgery again highlighting how difficult it may be to accurately diagnose these patients. Finally, our technique was associated with no immediate postoperative complications. Only one patient was required to return to the operating room for redo minimally invasive decompression and fusion. Ultimately, this approach demonstrates advantages compared to previous techniques by the lack of bony facet removal, thus maintaining facet integrity and minimizing the chance of instability at that segment. Furthermore, this technique differs from the traditional far lateral as bone removal is minimized to reduce the chance of nerve root injury and in some cases, the nerve root was simply decompressed by removing the intertransverse ligament with less aggressive attempts at disc removal. Last, this technique is performed MIS through a tube, as traditional far lateral is performed with a small, open, paramedian incision with the use of the operating microscope. The future of spine surgery includes the use of enabling technologies and even more minimally invasive and ergonomic options may exist for far lateral microdiscectomy. These include ultra-minimally invasive techniques like endoscopic discectomy and the use of the exoscope to replace the operating microscope [30].

Limitations

This is a single-institution, retrospective study, and, as such, there are limitations to be noted. We relied on current procedural technology (CPT) 4 coding to identify patients undergoing MIS far lateral lumbar discectomy. CPT-4 codes alone cannot capture the many procedural variables that may influence intraoperative and postoperative adverse events. Furthermore, this study is a single-institution summary of the author’s experience with a specific technique for a cohort of patients and did not seek to compare competing interventions. Another limitation was the small sample size of patients that underwent this procedure over the 10-year time period. Another limitation was the mean postoperative follow-up time of 4.3 ± 2.7 months following decompression which may not capture the return of symptoms (radicular or back pain), but we posit the majority of postdiscectomy symptoms would present themselves early and within this timeframe. It should be noted that complete cohort radiographic results and patient-reported quality-of-life outcomes were beyond the scope of the current study.

## Conclusions

MIS far lateral discectomy can be completed successfully with minimal to no bony drilling while maintaining a high rate of symptomatic improvement for patients. Our modification of this minimally invasive technique may reduce postoperative pain and the potential risk of iatrogenic spinal instability without sacrificing symptomatic relief.
